# Enhanced electrochemical discharge of Li-ion batteries for safe recycling†

**DOI:** 10.1039/d4se00125g

**Published:** 2024-05-28

**Authors:** Neha Garg, Simo Pekkinen, Eduardo Martínez González, Rodrigo Serna-Guerrero, Pekka Peljo, Annukka Santasalo-Aarnio

**Affiliations:** a Research group of Energy Conversion and Systems, Department of Mechanical Engineering, School of Engineering, Aalto University PO Box 14400 Aalto FI-00076 Finland neha.garg@aalto.fi; b Research group of Mineral Processing and Recycling, Department of Chemical Engineering and Metallurgy, School of Chemical Engineering, Aalto University PO Box 16200 Aalto FI-00076 Finland; c Research Group of Battery Materials and Technologies, Department of Mechanical and Materials Engineering, Faculty of Technology, University of Turku Turun Yliopisto FI-20014 Finland

## Abstract

The recycling of spent lithium-ion batteries (LIBs) is crucial to sustainably manage resources and protect the environment as the use of portable electronics and electric vehicles (EVs) increases. However, the safe recycling of spent LIBs is challenging, as they often contain residual energy. Left untreated, this can trigger a thermal runaway and result in disasters during the recycling process. For efficient recycling, it is important to withdraw any leftover energy from LIBs, regardless of the processing method that follows the discharge. The electrochemical discharge method is a quick and inexpensive method to eliminate this hazard. This method works by immersing batteries in an aqueous inorganic salt solution to discharge LIBs completely and efficiently. Previously, research focus has been on different inorganic salt solutions that release toxic or flammable gaseous products during discharge. In contrast, we present an entirely new approach for electrochemical discharge – the utilization of an Fe(ii)–Fe(iii) redox couple electrolyte. We show that this medium can be used for efficient LIB deep discharge to a voltage of 2.0 V after rebound, a level that is low enough for safe discharge. To accomplish this, periodic discharge methods were used. In addition, no corrosion on the battery casing was observed. The pH behavior at the poles was also investigated, and it was found that without convection, gas evolution during discharge cannot be avoided. Finally, it was discovered that the battery casing material plays a vital role in electrochemical discharge, and its industrial standardization would facilitate efficient recycling.

## Abstract 2

The safe recycling of spent LIBs is challenging, as they often contain residual energy. Left untreated, this can trigger a thermal runaway and result in disaster during the recycling process. Electrochemical discharge method is an easy and inexpensive method to eliminate this hazard.

## Graphical abstract

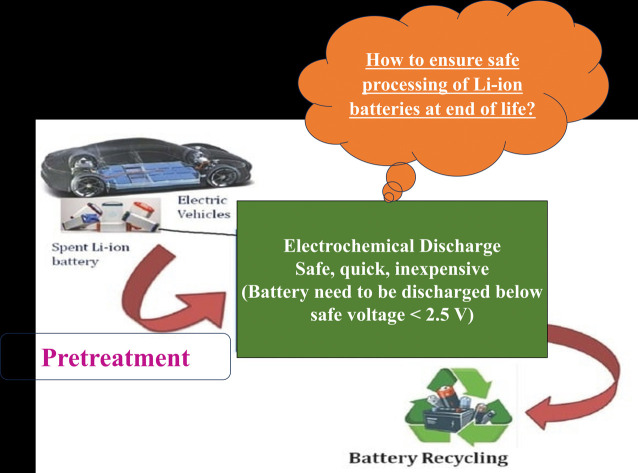

## Introduction

The growing awareness of climate change has led to efforts to reduce CO_2_ emissions, mainly caused by the use of fossil fuels.^1^ As the world transitions to emission-free electrification, batteries are becoming a vital energy storage tool in stationary and electric vehicle applications. Due to their high energy density and good recharge capability, lithium-ion batteries (LIBs) have become the frontrunner of electrical energy storage options for future energy systems.^2^ The use of LIBs has increased since their initial discovery and then amplified exponentially after they were first commercialized by Sony in 1991, resulting in the awarding of the Nobel Prize in 2019:^3–6^ they are in our pockets, in our backpacks, in our household equipment, in our cars and solar panel systems, *etc.*^7,8^ There is a massive demand for LIBs, and the importance of LIBs as power sources is reflected by the steady increase in their production rate and their continuously growing market share. Especially the increase in the demand for green transport has led to an increase in the number of electrical vehicles (EVs) that almost exclusively run on LIBs. It's been estimated that the global LIB demand is expected to rise from 300 GWh to 2000 GWh per year over the next decade, with a significant contribution from electric passenger vehicles.^9^ According to one survey, in February 2019 there were over 5.6 million EVs in the world, and it is anticipated that by 2040, 58% of all cars sold worldwide will be EVs.^10,11^ Recent years have seen a rapid increase in the demand for EVs; in 2021, there were about 5.5 million electric cars on European roads – more than three times the stock of 2019, with the global EV fleet expected to reach 7.5 million by 2030.^2,12^ Consequently, the annual production capacity of LIB cathode materials can be on the order of at least 40 GWh per year, or 200 000 tons.^13,14^ It is estimated that the total mass of LIBs reaching the end of life in 2030 will be more than 250 M tonnes.^15^ Spent LIBs contain critical materials such as cobalt (5–20%), nickel (5–10%), lithium (5–7%) and other metals like copper, aluminium, iron and manganese (5–10%),^16,17^ Therefore with this scale of production and the number of batteries that will be retired, it important to ensure long-term supply chain sustainability.^18^ Therefore, automakers and battery industrialists have expressed keen interest in a range of end-of-life scenarios for spent packs and battery cells, including the recovery of materials from used battery packs.^19,20^ Also from government policies, in the European Green Deal 2020, a legislative proposal was submitted by the European Commission to replace the 2006 Battery Directive, which proposed new collection targets for waste portable batteries (excluding batteries for light means of transport, *e.g.*, e-bikes) of 45% by 2023, 65% by 2025, and 70% by 2030.^21^ Thus, the growing supply of end-of-life LIBs meets a corresponding industrial demand for efficient and safe recycling technologies.

At the recycling facilities when spent LIBs enter, they still have residual energy that causes fire hazards because of the leftover charge in LIBs, also the organic electrolyte reacts to produce harmful toxic vapours during the recycling process. During the crushing stage, there is also a risk of explosion due to the possibility of a short-circuit between the cathode and the anode, which releases an enormous amount of energy.^16,17^ Therefore, collecting, storing, and transporting LIB waste is a source of fire hazards at the collection points and recycling facilities.^22–25^ As a result, to ensure safe and efficient recycling, the excess energy needs to be removed from the battery before dismantling. To overcome these hurdles, we urgently need reliable, fast, and cost-efficient technical solutions to ensure the safe discharge of waste batteries. The recycling of spent LIBs mainly consists of two steps, pre-treatment of spent batteries and the recovery of components. Pre-treatment includes deactivation or discharging, dismantling, crushing, and component separation,^26,27^ while the recovery of the components comprises hydrometallurgical, pyrometallurgical and direct methods, in which leaching, separation, extraction, and chemical/electrochemical precipitation are usually involved.^28^ For these recycling steps, materials must be separated from each other either by crushing and liberation to pure streams or through direct recycling, where the cells are opened, and different components are separated from each other without a crushing step. For either of any approaches, it is essential that the cells do not contain any excess energy that can pose a threat to life and equipment. To open the cells or to crush them, the state of charge (SOC) should preferably be below 0% (corresponding to a voltage below 2.5 V).^16,29^ To reduce the violent release of energy during the pre-treatment process, different discharging techniques such as freezing the batteries with liquid nitrogen before crushing^30^ or recycling them in an inert atmosphere^31,32^ have been proposed; however, due to the addition of further use of materials, these methods have been found expensive.^23^ The other technique is a conductive powder buried discharge or a non-electrochemical discharge in a conducting powder; however, the use of conductive powder can create additional waste and environmental concerns.^33^ A recent study also shows the effect of temperature and the C rate on the discharging of spent 18 650 LIBs.^34^ Among all these solutions, electrochemical discharge of spent batteries has been accepted as a stout and straightforward discharging step to address these potential hazards.

Prior to 2017, the literature about the electrochemical discharge of LIBs was centred around the simplistic statement of: “batteries can be discharged in salt solutions”^35–43^ where the salt solutions were mainly NaCl or Na_2_SO_4_. However, there were no experimental data available in the scientific literature to prove the validity of this statement. We have conducted experiments to test these claims and observed rapid corrosion of the LIB poles in various aqueous salt solutions.^44^ Later, these findings were independently confirmed by other research groups.^45–48^ The fast corrosion, particularly in NaCl solutions, inhibits both the electron transfer and the accurate measurement of voltage, while the batteries remain charged. The most alarming aspect is that in many recycling review papers, the dischargeability of LIBs in salt solutions is accepted as a fact without any critical evaluation.^29,49–51^ Since 2019 a few reviews have taken a more nuanced stance, broadly concluding that submerging batteries in discharge media should be further studied.^52–54^ In early studies, only the battery discharge voltage immediately after the removal from the electrolyte was reported. However, a study by Nembhard *et al.* that recorded the battery voltage trend for a longer time observed that the batteries settled at a higher voltage level over the course of the experiments.^48^ This voltage increase is called voltage rebound in electrical engineering and voltage relaxation in electrochemistry. For clarity, it will be called voltage recovery in this study. In recent years, the occurrence of voltage recovery has been acknowledged, but the underpinning phenomena are still not fully understood.^55,56^ Electrical engineering research on voltage recovery has focused on exploiting it for extending battery operation,^57,58^ whereas the electrochemical community has studied voltage recovery mainly in the voltage range above 2.5 V (>0% SOC) for State-Of-Health (SOH) analyses.^59^ In our previous research, we have systematically studied the suitability of different inorganic salts (NaCl, K_2_CO_3_, NH_4_CO_3_, *etc.*) to discharge LIBs, and we found that these solutions cannot discharge batteries low enough for recycling purposes after voltage recovery is considered.^60,61^ Another disadvantage of using inorganic salts is that they are most likely consumed in the reaction, leading to further material waste in addition to the long discharge time (several days).

In this work, we introduce an entirely new approach for the electrochemical discharge of LIBs: we utilize a well-known iron-based ferro/ferricyanide redox couple to enable oxidation and reduction reactions at the LIB poles to consume the electrons from the batteries. With this type of system, it could be possible to find electrolyte media that would not produce toxic or flammable gases. Additionally, if a system with minimal side reactions could be discovered, any significant consumption of the electrolyte species could be avoided. In our research, we have found that redox couples are more efficient than just individual inorganic salts, and they can provide non-corrosive discharge media for batteries that have a corrosion tolerant stainless-steel casing.

## Materials and methods

To study the applicability of the methodology for a larger variety of battery chemistries, we selected commercially available Li-ion batteries with differing chemistries (LCO, NCA and NMC Li-ion batteries). We further narrowed our focus to three providers that are available for consumer electronics. We used three commercially available LIBs: Biltema ICR18 650 (Sweden, capacity 2950 mA h) with a protective circuit, Panasonic Cameron Sino CSNCR18 650B (China, capacity 3250 mA h) without a protective circuit and Ansmann Li-ion battery 18 650 (Germany, capacity 3500 mA h) with a protective circuit, the properties of which are listed in Table 1. The batteries with a protective circuit contain an electronic circuit in their shell that prevents the battery from being charged and discharged too quickly and keeps the battery's charge within a safe range. The Panasonic battery also has junction plates at its poles, increasing the conductive area shared with the solution and thus affecting its discharge properties. The batteries used in the experiments were all new and unused to ensure constant SOH and starting voltage.

**Table 1:** Properties of the commercial batteries used in this study

	BILTEMA ICR18 650 (LCO)	Panasonic Cameron Sino CS-NCR 18 650B (NCA)	Ansmann Li-ion 18 650 (NMC)
Cathode material	LiCoO_2_	LiNi_1−*x*−*y*_Co_*x*_Al_*y*_O_2_	LiNi_1−*x*−*y*_Co_*x*_Mn_*y*_O_2_
Anode material	Graphite	Graphite	Graphite
Nominal voltage	3.7 V	3.7 V	3.6 V
Max voltage	4.35	4.2	4.2
Capacity	2950 mA h	3250 mA h	3500 mA h
Protective circuit	Yes	No	Yes
Other		Junction plates	

To study the effect of the electrolyte conductivity on the reaction rate, multiple salt concentration levels were used, with the aim of obtaining the lowest possible discharge voltage and the fastest discharge time. The salts used were potassium hexacyanoferrate (ii) trihydrate (VWR International, 99.9%) and potassium hexacyanoferrate (iii) (VWR International, 99.8%). Three different concentrations *i.e.*, 2.5, 5 and 10 wt% of solutions were prepared with de-ionized water (<0.5 μS cm^−1^). The solutions were prepared by measuring a given weight of crystalline potassium hexacyanoferrite and potassium hexacyanoferrate salts with an accurate scale and dissolving them into a given volume of de-ionized water to form solutions with different concentrations (w/v%). Saturated solutions were prepared by measuring a given volume of de-ionized water and adding both salts (by the same weight) until the crystals no longer dissolved. Then, the saturated solution was moved to another beaker to remove excess salt. When preparing all solutions, stirring was used to speed up the dissolving process. Since the experiments spanned several days, evaporation of water occurred, and additional de-ionized water was added whenever necessary to maintain the concentration constant.

To measure the voltage of the battery during the electrochemical discharge of the battery, a digital voltmeter (Fluke 87V TRMS Industrial Multimeter) was used, because it has provided results with higher accuracy for this purpose, as presented in detail in our previous publication. Powder X-ray diffraction (XRD) was carried out using a Cu source Panalytical X'Pert Pro diffractometer fitted with a monochromator operated at 45 kV and 40 mA (K_α1_, *λ* = 1.54056 Å). For elemental compositions, we have used a portable XRF gun (Oxford Instruments, X-MET 5000, Abingdon, United Kingdom). In this study, we discuss the single cell of spent 18 650 LIBs, and the discharge has been quite slow and has been taking a long time and it is not generating heat. But for the large EV batteries with high energy capacity (in A h), the discharge current and generated heat could be high. In that case, we need to make sure that the electrochemical battery discharges in the safe temperature range. For example, we should use a cooling system and monitor the temperature of the battery during its electrochemical discharge.

### Methodology to measure the battery voltage during electrochemical discharge

**Fig. 1:**
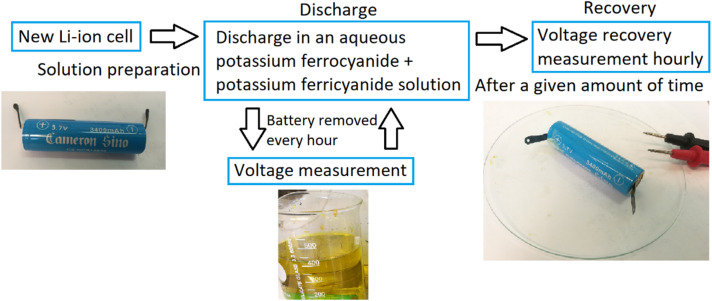
The experimental measurement setup and procedure for the battery voltage behaviour.

Before the experiments, the initial voltage of the battery was recorded. To start the experiments, the batteries were submerged in a glass beaker containing the electrolyte. During the discharge phase of the experiments, the voltage of the battery was measured by lifting the battery from the solution, rinsing the poles with de-ionized water, and then reading the open circuit voltage (OCV). Once the voltage was measured, the battery was submerged back into the electrolyte. The batteries were discharged for a fixed period or until significant corrosion on the casings occurred and the experiment was halted. A simplified flowchart of the procedure is presented in Fig. 1.

When using the periodic discharge method, the battery was first discharged for 48 h, and then removed from the electrolyte, dried, and left for the day while measuring the voltage recovery hourly. In the afternoon, around 8 h after its removal from the electrolyte, the battery was once again submerged in the electrolyte and discharged overnight. The next morning, it was once again removed for the day and submerged in the afternoon. This was repeated until roughly 100 hours were reached. The batteries were kept inside a fume cabinet on a glass plate and the voltage was measured periodically to monitor the voltage recovery of the batteries until equilibrium was reached.

### Methodology to measure the battery half-cell voltage and discharge current during electrochemical discharge

**Fig. 2:**
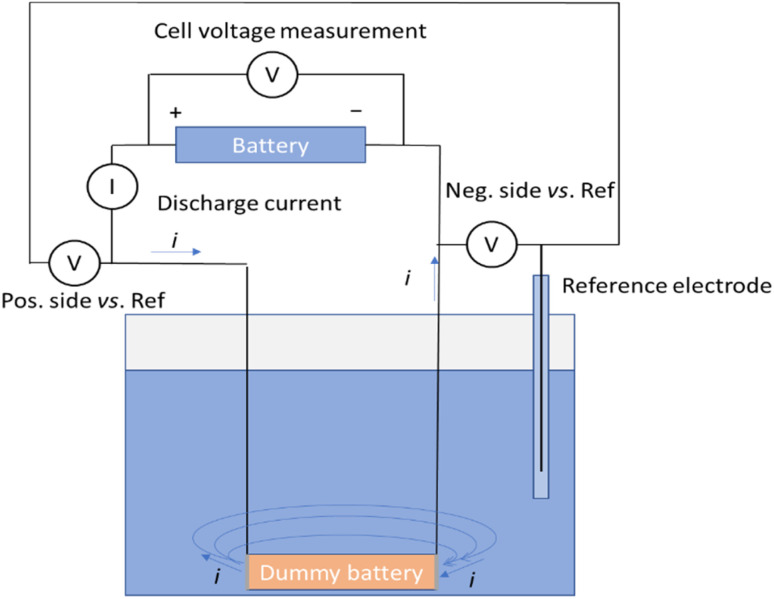
Schematic of the measurement system.

A measurement system illustrated in Fig. 2 was developed to measure the discharge current, voltage of the battery and voltages of the negative and positive terminals of the battery *vs.* an Ag/AgCl/3 M KCl reference electrode. In this set-up, a dummy battery with the same dimensions as the actual battery was prepared, with terminals recovered from a disassembled battery [Fig. 1 and ESI†].

Wires were soldered to connect to the terminals. The wires were then protected from exposure to the electrolyte with heat shrink tubing. A battery cycler (Landt Instruments, G340A, China) was utilized to record the battery voltage, as well as the voltages of the terminals against the reference electrode, and the fourth channel of the cycler was used to record the current passing through the circuit during the chemical discharge. Fig. 2 shows the schematic of the measurement system allowing simultaneous recording of the discharge current, the battery voltage, and the potentials of the terminals *versus* a reference electrode. The dummy battery of similar dimensions to the real one has two metallic terminals, where the electrochemical reactions take place. Current flows from the battery through the current meter into the dummy battery terminal and is transferred into the electrolyte in an electrochemical reaction. Then the current flows through the electrolyte solution to the other terminal and is transferred in another electrochemical reaction into the metal plate, where it will flow through the wire to the negative end of the battery.

## Results and discussion

### Electrochemical discharge of LIBs in an Fe(ii)–Fe(iii) redox couple electrolyte

In electrochemical discharge, the batteries are typically submerged into an aqueous salt solution that acts as a primitive resistor or controlled short-circuit to discharge the batteries. When aqueous electrolytes are used, the water-splitting reaction is always one possible reaction for discharging the batteries.^60–62^ Although the thermodynamic voltage required for the water-splitting reaction is 1.23 V, in practice higher applied voltage is required to overcome the overpotentials at the electrodes (1.65–1.9 V). This in turn precludes discharging to low enough voltages for safe recycling. Water splitting reactions at the anode and cathode are as follows:^62,63^

Anode: water oxidation reaction (oxygen evolution reaction) under alkaline conditions

1 4OH^−^ → O_2_ + 4e^−^ + 2H_2_, (*E*^0^ = 0.401 V *vs.* SHE at pH 14)

Cathode: water reduction reaction under alkaline conditions

2 4H_2_O + 4e^−^ → 2H_2_ + 4OH^−^, (*E*^0^ = −0.828 V *vs.* SHE at pH 14)

Net reaction:

3 2H_2_O → 2H_2_ + O_2_, (*E* = 1.23 V)

**Fig. 3:**
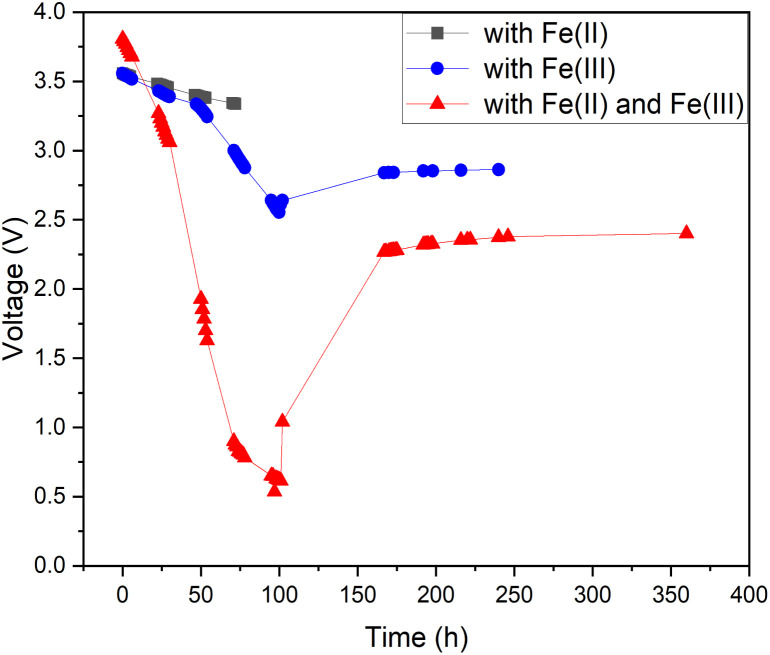
Internal electrochemical discharge of Panasonic LIBs in individual 5 wt% potassium hexacyanoferrate(ii) and 5 wt% potassium hexacyanoferrite(iii) and in an electrolyte containing a 5 wt% solution of potassium hexacyanoferrate and potassium hexacyanoferrite.

As pure water has a low conductivity, inorganic salts can be added to aqueous solutions to increase their conductivity.^44,47^ This also enables other chemical reactions to occur, some of which have lower onset potentials than the water-splitting reaction.^45,46^ Therefore, it is possible to discover a reaction with a lower theoretical voltage than water splitting to achieve deeper discharge of LIBs. One possible option would be inorganic redox couples that could undergo a cyclic reaction and would not be consumed during discharge. To test this hypothesis, we chose a commonly used redox salt pair: potassium hexacyanoferrate and potassium hexacyanoferrite. We also tested both individual salt electrolytes as a comparison to the behaviour of their mixture (redox couple). The results of this comparison are presented in Fig. 3. As can be seen, individual salts were not efficient for LIB discharge: the Fe(ii) electrolyte did not discharge the LIB below 3.3 V (in 72 hours) and in the Fe(iii) electrolyte, the battery voltage reached roughly 2.5 V after 100 h, followed by a recovery up to 2.8 V. In comparison, LIBs in the electrolyte containing both Fe(ii) and Fe(iii) salts reached a significantly lower voltage of 0.7 V after 96 h. Afterwards, voltage recovery plateaued at 2.4 V. These results confirm that combining the two salts into a redox pair significantly improves the discharge properties of the electrolyte and offers the possibility of deep discharge of LIBs.

**Fig. 4:**
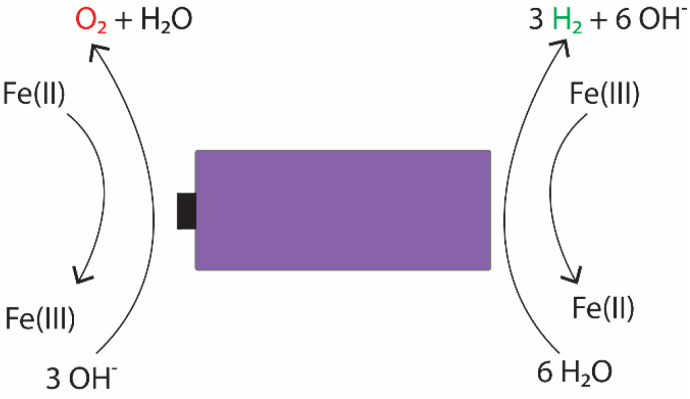
Possible electrochemical reactions occurring in the electrochemical discharge of LIBs in an electrolyte containing potassium hexacyanoferrate and potassium hexacyanoferrate salts.

For the individual salts, gas evolution was observed on both poles of the battery instantly when the battery was submerged into the electrolyte. The gases were most likely hydrogen and oxygen formed through the water splitting reaction. This gas evolution slowed down significantly as the voltage decreased and stopped completely once the voltage reached below 2.5 V, which would imply that the water splitting reaction could be the most dominant reaction consuming the electrons. This is a very similar behaviour that has been observed for several other individual inorganic salts.^47,48,60,61^ Meanwhile, when a redox couple was utilized, the voltage decreased below the theoretical limit of water splitting (1.23 V), meaning that another electron consuming reaction must have taken place (Fig. 3). While mixing Fe(ii) and Fe(iii) salts together, both oxidation and reduction reactions of iron occur simultaneously at the positive and negative electrodes (Fig. 4). These redox reactions will compete with the water splitting reaction and are capable of efficiently consuming the electrochemical energy from the battery, even at lower battery voltage levels – the redox potential of ferro/ferricyanide oxidation/reduction is *E*^0^ = 0.358 V *vs.* SHE V.^64^

**Fig. 5:**
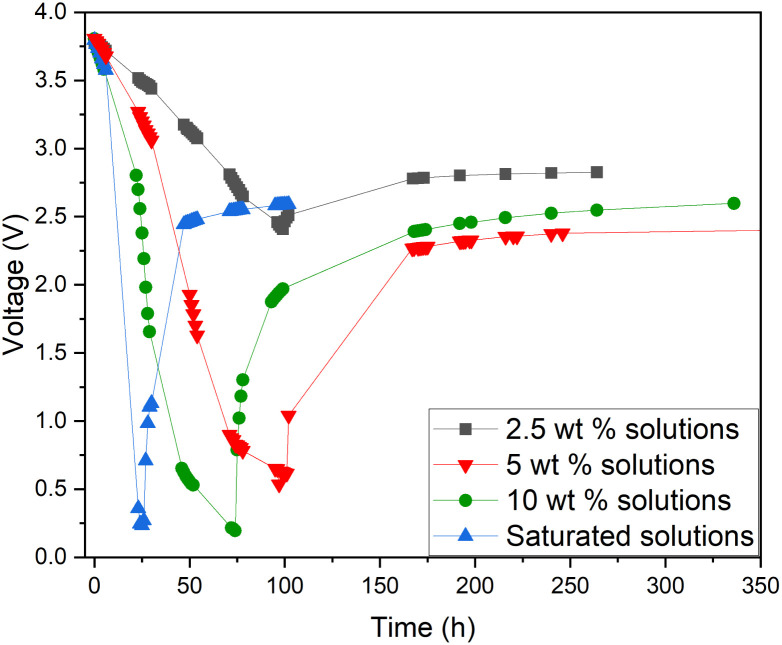
Electrochemical discharge of a Panasonic LIB in a redox electrolyte containing potassium hexacyanoferrate(ii) and potassium hexacyanoferrite(iii) in different concentrations from 2.5 wt%, 5 wt%, 10 wt% to saturation solution.

**Fig. 6:**
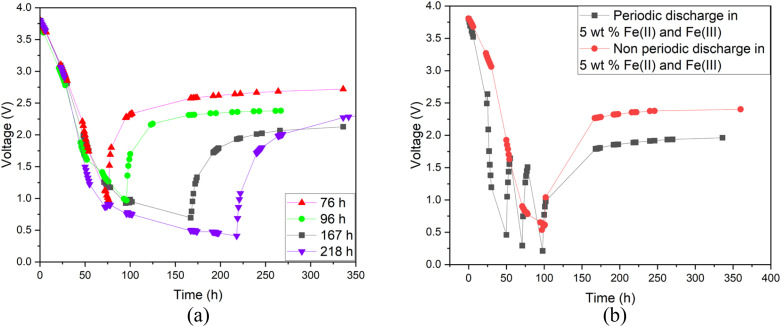
(a) Discharge behaviour of Panasonic LIBs with varying discharge times. (b) Comparison of periodic and non-periodic discharge of a Panasonic LIB; both experiments were conducted in a redox electrolyte containing a 5 wt% solution of potassium hexacyanoferrate and potassium hexacyanoferrite.

Additionally, a small amount of solid product was detected. This would suggest that a side reaction, most likely the formation of Prussian Blue (as evidenced by its blue colour), occurs during electrochemical discharge (ESI, Fig. S1†) and is further discussed. As this side reaction consumes the reactants from the electrolyte, we aimed to fine-tune the discharge conditions to reduce such unwanted reactions. First, we studied the impact of redox electrolyte concentration that was varied from 2.5 wt% up to saturated solutions (2.5 wt%, 5 wt%, 10 wt% and saturated solution) and the corresponding results are presented in Fig. 5. The discharge was terminated when a voltage of 0.5 V or lower was obtained, but the cells were monitored for voltage recovery up to a stable value. Fig. 5 concludes that a low concentration (2.5 wt%) is not enough to support the efficient discharge of the LIBs, as the battery voltages decreased only to 2.5 V, and it recovered to more than 2.8 V. Most likely, water splitting is still the dominant reaction at this concentration, and deep discharge is not obtained. With a higher concentration, at 10 wt%, the voltage decreased to 0.2 V, but it recovered to 2.6 V. However, at an electrolyte concentration of 5 wt%, voltage decreased to 0.5 V, and only recovered to roughly 2.4 V, which is below the 0% SOC (<2.5 V) required for safe battery processing. While using saturated solutions of Fe(ii) and Fe(iii) salts, voltage dropped very fast (within 24 h) to around 0.2 V, but it also recovered quickly to almost 2.6 V. This is typical for LIB voltage recovery behaviour, as faster discharge characteristically leads to higher recovery voltage, as reported previously in the case of aqueous carbonate electrolytes.^47^ Overall, it can be concluded that when the electrolyte concentration was 5 wt% or above, the impact of the redox couple was clear and deep discharge was attained. The higher the electrolyte concentration, the faster the discharge rate, an observation consistent with findings presented in earlier studies.^46^ After finding that the 5 wt% electrolyte achieved the lowest voltage recovery after deep discharge, we continued our discharge experiments with this concentration to further optimize the process. To investigate the effect of discharge time on the recovery voltage, discharge time within the electrolyte was varied and the results are presented in Fig. 6(a). Additionally, a concept introduced in our previous work for controlled discharge, periodic discharge, was shown to further decrease the recovery voltage, even with lower discharge times.^65^ Therefore, we tested if periodic discharge could be implemented to further reduce the discharge time without simultaneously increasing the voltage recovery. Fig. 6(a) shows a very similar discharge behaviour for all four batteries in the beginning of the experiments, which reinforces the repeatability of these studies. The differences begin to show once the batteries were removed from the electrolyte at different discharge times; the first three shorter experiments showed a decreasing trend in recovery voltage. While the longest discharge time (218 h) did lead to the lowest discharge voltage value, the recovery voltage was similar to that of the 167 h discharge time experiment. Therefore, there is no additional advantage to exceeding 167 h discharge time. In Fig. 6(b), we present a periodic discharge study of a LIB in a redox coupled electrolyte. This was implemented by reintroducing the batteries into the electrolyte after an 8-hour voltage recovery time outside the electrolyte. It can be observed that while the battery was submerged directly into the electrolyte, it discharged to 0.5 V in 100 h, but when the battery was removed from the electrolyte, the recovery voltage increased to 2.4 V. However, when the controlled periodic discharge method was used, first when the battery was submerged into the electrolyte, it discharged to 0.5 V within 50 h, and then when the battery was removed from the solution for 8 h, the voltage recovered to 1.7 V. This pattern was then repeated two more times. During the first repeat, the battery was discharged to 0.4 V and recovered to 1.5 V, and during the second repeat it discharged to almost 0.2 V and recovered to 1 V in 100 h. After this, the battery was allowed to recover in air, resulting in a voltage recovery to 1.7 V. This comparative study confirms that with periodic discharge, it is possible to attain a voltage lower than 2.0 V. This breakthrough confirms that it is possible to find conditions in which LIBs can be discharged in inorganic electrolytes below 2 V, paving the way for the safe processing of spent batteries.

### LIB battery half-cell experiments in an Fe(ii)–Fe(iii) redox couple electrolyte

**Fig. 7:**
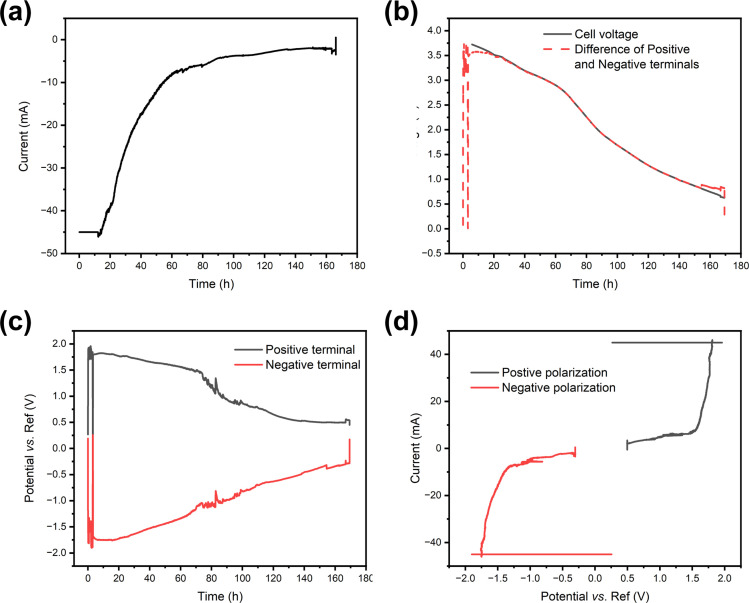
Electrochemical discharge of a Panasonic battery, in a 5 wt% potassium hexacyanoferrate and potassium hexacyanoferrate electrolyte. (a) Measured discharge current, (b) cell voltage, (c) half-cell voltages measured against an Ag/AgCl reference electrode and (d) current plotted *vs.* the potential of the terminal.

As the redox couple electrolytes show efficient discharge behaviour, we wanted to understand better what reactions take place at the terminals of the electrodes, and how those reactions affect the pH of the system. For this purpose, a measurement system described in Fig. 2 was employed, with a Panasonic LIB in a redox electrolyte containing a 5 wt% solution of potassium hexacyanoferrate and potassium hexacyanoferrite. Fig. 7 shows the current, cell voltage and half-cell voltages measured against an Ag/AgCl reference electrode. Fig. 7a shows that in the beginning the current flow was limited to −45 mA and then allowed to reach short circuit current limited by the electrochemical reactions. The decrease in current shows exponential decay until a steady state current of *ca.* 5 mA is reached. Fig. 7b shows that the cell voltage decreases rather linearly, in agreement with the earlier experiments. As expected, the cell voltage measured from the cell and that evaluated as the difference of the two half-cell voltages agree, except in the beginning. Fig. 7c shows that the positive terminal reaches a voltage of *ca.* 1.8 V *vs.* the reference electrode, while the negative terminal reaches voltages of −1.7 to −1.8 *vs.* the reference electrode almost immediately after the onset of the chemical discharge, and gas evolution is observed. These measurements show that both the potentials of the half-cells go to regions where hydrogen and oxygen evolution reactions take place, *i.e.* the limiting currents for Fe(ii) oxidation at the positive terminal and Fe(iii) reduction at the negative terminal are exceeded. Thermodynamically, the limits for oxygen and hydrogen evolution at pH 7 are *ca.* 0.61 V and −0.62 V *vs.* Ag/AgCl, respectively. As both reactions require very good electrocatalysts to take place close to the thermodynamic limit, in practice high overpotentials are expected on the terminals. The measured current can be plotted against the measured potential to allow the evaluation of the polarization curves in Fig. 7d that shows a sharp increase in current at *ca.* 1.6 V *vs.* Ag/AgCl, and a sharp decrease at *ca.* −1.3 V *vs.* Ag/AgCl. Therefore, an overpotential of *ca.* 1 V is required to realize significant oxygen evolution at the terminal material. Hydrogen evolution is slightly easier, requiring an overpotential of only −0.7 V. The onset of significant oxygen evolution requires a potential of *ca.* 1.5 V *vs.* an Ag/AgCl reference, while hydrogen evolution requires *ca.* −1.3 V *vs.* an Ag/AgCl reference, in line with what has been reported earlier on stainless steel.^66^

**Fig. 8:**
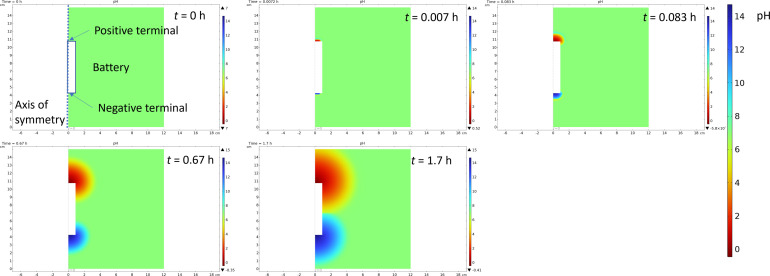
Snapshots of the simulation of pH change during the chemical discharge of the Li-ion battery.

Finite element simulations described in the ESI (Fig. S2†) were done to evaluate how a large portion of the current is coming from the water splitting reaction, and how this affects the pH of the system. For this purpose, the battery was considered to be surrounded in an electrolyte containing a 5 wt% solution of both ferri- and ferrocyanide (170 mM Fe(iii) and 132 mM Fe(ii)) to allow simulations in 2D axis symmetry. Simulations show that already after 100 s less than 10% of the current is coming from the reactions with the Fe(iii)/Fe(ii) electrolyte, and the rest is coming from the gas evolution. The drawback of gas evolution reactions is that the pH of the solution changes. It is well known that ferricyanide and ferrocyanide are not stable under acidic conditions, generating highly toxic HCN.^67^ Additionally, ferricyanide is not stable under highly alkaline (pH 14) conditions.^68^ Finite element simulations were used to investigate if these extreme pH values could be reached. Now, the experimentally measured current was used as an input value for the simulations, and Fe(ii) oxidation at the positive terminal and Fe(iii) reduction at the negative terminal were assumed to take place at the diffusion limited rate. The difference between the currents was assigned to gas evolution, consuming, or generating protons according to eqn (1) and (2). The simulations show that the changes in the pH are very drastic, with both terminals experiencing highly acidic or highly alkaline conditions already after 5 s. The simulated change in the pH is shown in Fig. 8.

Gas evolution will cause convection due to the generated bubbles, so mass transfer will be enhanced, and this was not considered in the simulations. But highly acidic pH simulated at the positive terminal suggests that the decomposition of iron complexes could lead to the generation of highly poisonous HCN gas. Moreover, if hydrogen and oxygen are generated simultaneously, the mixing of these gases can lead to an explosive mixture if ventilation from the system is not provided. Simulations were carried out with different concentrations to evaluate if gas evolution could be avoided. But even in 5 M solutions more than 60% of the current would be used for gas evolution reactions. Therefore, significant convection would be required to increase the mass transfer rate of the mediator, increasing the limiting current and allowing safe discharge of the batteries.

### Studying the effect of casing materials of different LIBs on discharge

**Fig. 9:**
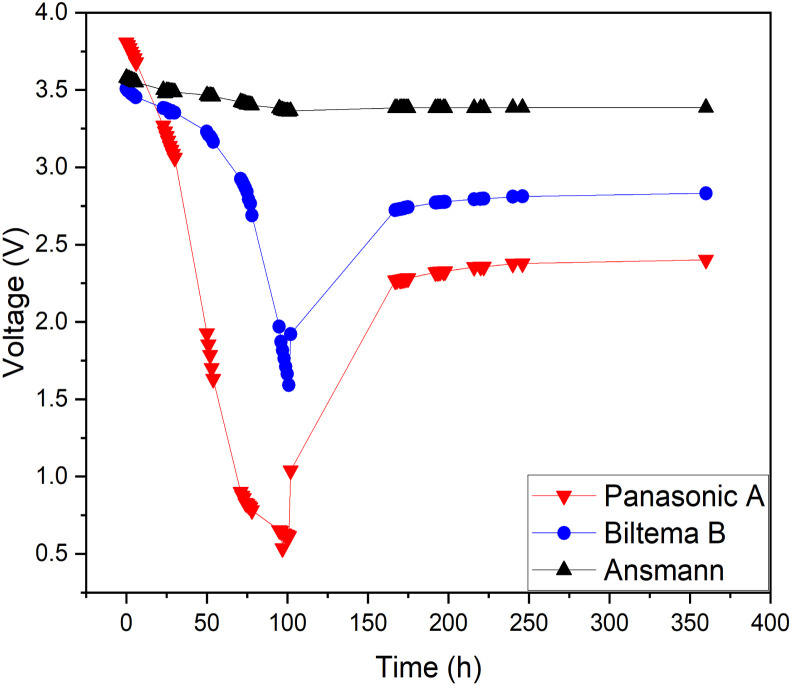
Discharge behaviour of different batteries in a redox electrolyte containing a 5 wt% solution of potassium hexacyanoferrate and potassium hexacyanoferrite.

**Fig. 10:**
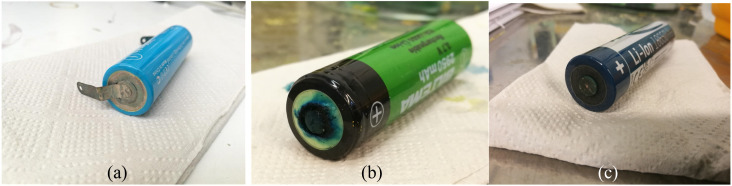
Corrosion of the battery poles after discharging experiments: (a) Panasonic battery with junction plates at the terminals, (b) Biltema battery, and (c) Ansmann battery.

So far, the investigations made for the electrochemical discharge by our group and others have only focused on the discharge of one single provider of the battery as there is no current standardization of battery materials. To investigate the relationship between different providers of LIBs and their discharging behavior towards redox coupled electrolytes, we have selected three different chemistries of LIBs (18 650 type) Panasonic-NCA, Biltema-LCO and Ansmann-NMC. Since all the batteries were 18 650-type with a similar nominal voltage, the SOC–voltage relationship was assumed to be the same for all batteries. Fig. 9 shows the discharge-recovery curves of the three different batteries from the three different providers in a 5 wt% redox coupled electrolyte with a discharge time of 100 h and the corresponding photos of the battery positive poles are shown in Fig. 10(a–c).

Fig. 9 clearly shows that the voltage trend of the Ansmann battery changed very little during the electrochemical discharge, decreasing only to 3.4 V from 3.6 V. This is due to the fast corrosion of the battery poles, which inhibited the electron transfer through the corroded oxide layer that can be observed in Fig. 10(c) as a black discoloration of the initially metallic pole. The Panasonic LIB discharged to 0.5 V and then reached a recovery voltage of roughly 2.4 V. The discharge of the Biltema battery was initially very slow, but after two days (50 h), it discharged rapidly to 1.6 V and after removal from the electrolyte, it reached a recovery voltage of 2.8 V.

**Fig. 11:**
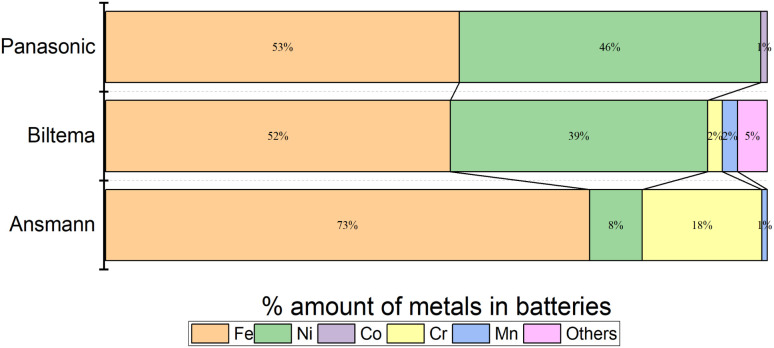
Elemental analysis of LIBs from different manufacturers: (a) Biltema, (b) Panasonic and (c) Ansmann, measured with an XRF.

Fig. 10 shows the images of the battery poles after 100 h of discharging in the aqueous electrolyte. We found that Ansmann and Biltema batteries experienced a higher corrosion rate at the poles in comparison to Panasonic batteries. The colour of the electrolyte solution has also been compared in Fig. S3, ESI.† The corrosion product was filtered away from the electrolyte and analysed by powder XRD, and these analysis results are given in Fig. S4, ESI.† The PXRD result confirmed our hypothesis that the residue found in the electrolyte is Prussian blue (PB, Fe(iii)_4_[Fe(ii)(CN)_6_]_3_) and unreacted potassium hexacyanoferrate(ii) trihydrate.^69,70^ On the battery pole, we detected the same compounds, and this additional layer caused an inhibition barrier that prevented the electrochemical discharge of the Ansmann battery. Therefore, this was not purely a corrosion on the casing phenomenon but a challenge that can arise in the side-reactions in the cyanide based redox couples. This underlines the importance of finding more suitable redox couples for efficient electrochemical discharge. However, corrosion is more likely to be related to the casing material of the batteries than to the battery chemistries, and there is no international standardization of battery casing materials. Therefore, we investigated the casing materials using an X-ray Fluorescence (XRF) spectrometer, and a rough elemental analysis of the casing materials for the three different batteries is shown in Fig. 11.

**Fig. 12:**
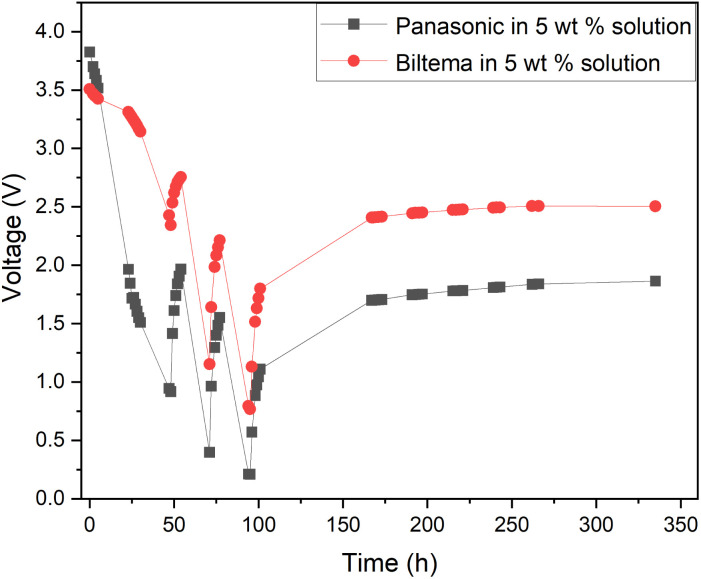
Comparison of the periodic discharge of Panasonic and Biltema LIBs in a redox electrolyte containing a 5 wt% solution of potassium hexacyanoferrate and potassium hexacyanoferrite.

The results from the XRF analysis (Fig. 11) revealed that there can be significant variation in the steel grade used for LIB casings from different providers. Ni-rich steels are used as a casing material for LIBs due to their excellent chemical resistance and corrosion protection. Our observations confirm that a lower Ni content provides less corrosion protection for the steel casing. Because it was observed that the Ansmann battery did not discharge properly due to corrosion that occurred at the casing and the side reaction product formation (Fig. 9 and 10), we proceeded with the Panasonic and Biltema batteries and performed a controlled periodic discharging experiment. The results are shown in Fig. 12.

Fig. 12 shows that both batteries exhibit similar trends during the periodic discharge experiments. With three cycles, the Panasonic battery discharged almost to 0.2 V and the final recovery reached only 1.7 V. On the other hand, the Biltema battery discharged only to 0.7 V in three cycles and settled on a final recovery voltage of a bit below 2.5 V. Here, the possible corrosion of the Biltema battery poles might have hindered the discharge of the batteries. Overall, this study confirms that with periodic discharge in a redox electrolyte, it is possible to deep discharge at least 2 different LIB chemistry types from two different manufacturers to a level that will be below 2.5 V after recovery. This is an interesting finding and will be the focus of our future study. As there were such large differences in the corrosion and discharge behaviour of the different batteries, a larger set of different casing materials and battery chemistries should be addressed, and this will be in focus in our further studies. However, it is important to highlight now that the battery casing material will have an impact on the pre-processing of these batteries, and this should be standardized on the field.

## Conclusions

To avoid the environmental, health and safety impacts of LIB recycling processes and to enhance the recovery rate of critical raw materials, it is essential to completely discharge the batteries prior to processing. Out of the different LIB discharge methods, electrochemical discharge is widely accepted among scientists as a robust method capable of the large-scale discharge of small batteries at a low cost. There have been previous attempts to discharge LIBs to a low voltage level in NaCl or other inorganic aqueous salt solutions as electrolytes, but either the electrochemical discharge has been very slow or the electrolyte has been very corrosive. Additionally, the voltage of LIBs has rebounded to high values after removing the batteries from the circuit (removal from the electrolyte). Therefore, there has not been an efficient, but corrosion free electrochemical discharge electrolyte available.

In this study, we showcased for the first time the possibility of utilizing electrochemical redox couple electrolytes to efficiently discharge LIBs to a low voltage level. By using a potassium hexacyanoferrate/ferrite electrolyte, it is possible to obtain a deep discharge of LIBs below 1 V within the electrolyte without major corrosion of the Panasonic battery casing. We studied the impact of different electrolyte concentrations where a 5 wt% electrolyte provided clear deep discharge but also the lowest rebound voltage (2.4 V) after removal from the electrolyte that is below the required safe recycling level, below 0% SOC to 2.5 V. We also found out that the discharge time above 167 h did not improve the discharge to any higher voltage, and thus with periodic discharge we were able to further reduce the rebound voltage of the Panasonic battery to 1.7 V without any sign of corrosion of the electrode poles.

As the results were extremely good, we wanted to further study the impact of a redox salt couple on the discharge phenomena as it competes with gas evolution reactions, and more detailed electrochemical experiments were conducted and the COMSOL model was used. In the best-case scenario, we could consume all the electrochemical energy from the battery by redox reactions in the electrolyte and could entirely avoid the formation of toxic or hazardous gases. Additionally, we would not consume the electrolyte species and reduce the need for electrolyte renewal. However, the result shows that gas evolution at both poles does take place even at high redox couple concentrations. It was identified that the mass transfer of the mediator was not high enough to prevent the gas evolution reactions. Thus, the situation could be enhanced by solution mixing, which will be the focus of a further study. Alternatively, batteries could be discharged first in inorganic aqueous salt solutions, followed by discharge in redox electrolytes. In this approach minimal gas evolution would take place in the second step, allowing further discharge of the batteries below 0% SOC without consumption of the electrolyte. Alternatively, redox couples stable in the whole pH range could be investigated. While this would be built as an industrial scale set-up, the water management incorporated with the electrochemical discharge must be taken into account properly. The Fe(ii)–Fe(iii) redox couple discharge medium is excellent for discharging the batteries, as the redox couples take part in the reduction and oxidation processes and are not consumed during the discharging of LIBs. Also, water splitting occurs slightly, which results in the evaporation of water from the solution and requires adding more water during the process. After the discharge steps, the discharged batteries would be lifted from the electrolyte and rinsed with water (this would replace the water that has been consumed in the electrochemical discharge and converted to gaseous products, H_2_ and O_2_). This would remove the possible salt residues from the batteries themselves. The electrolyte solution is re-useable in the process and when a batch of batteries is handled, it can be directly used for the discharge of the second batch. Casing corrosion must be entirely avoided to also ensure that the electrolyte solution remains uncontaminated by the corrosion products. It will also be important to further study if some minor salt residues will impact the hydrometallurgical processing of the batteries at a later stage.

As many electrochemical discharge studies have been conducted with only one type of battery, we wanted to extend the study to different LIB chemistries and selected three different chemistries from different providers. With these tests, we discovered that even more than the LIB chemistry itself, the casing steel material grade has a significant impact on the battery discharging behaviour. The results imply that low Ni-content stainless steels are not suited for electrochemical discharge in an aqueous environment. Therefore, we would propose a more comprehensive study of the minimum level of Ni content in the battery casing, which will determine the steel quality that is suitable for aqueous electrolyte discharge. These findings should be brought to the attention of the battery industry for the standardization of casing materials. This would enable electrochemical discharge as one of the pre-processing methods for all future small-scale batteries. This study makes our research path clear to provide the solution of giving a non-corrosive discharging medium for specific Li-ion batteries; however, for industrial application, this study needs further research because of the possible formation of toxic HCN. In summary, our demonstration of a non-corrosive deep discharge electrolyte along with our findings regarding the impact of casing materials and processing parameters paves the way for scalable battery discharge methods for safe recycling. Overall, with the two successful, low corrosion on the casing experiments, LIB chemistry has some impact on the discharge behaviour of the battery in electrochemical discharge and this needs to be addressed in more detail.

## Conflicts of interest

There are no conflicts to declare.

## Supplementary Material

SE-008-D4SE00125G-s001
