# Supplementary material for: Enhanced electrochemical discharge of Li-ion batteries for safe recycling
Source: Sustain Energy Fuels. 2024 May 28;8(12):2777–88. doi: 10.1039/d4se00125g (PMC11165673; doi:10.1039/d4se00125g)
Supplement: SE-008-D4SE00125G-s001 [file SE-008-D4SE00125G-s001.pdf]

Electronic Supplementary Material (ESI) for Sustainable Energy & Fuels.
This journal is © The Royal Society of Chemistry 2024

Supporting Information (SI)

Enhanced electrochemical discharge of Li-ion batteries for safe recycling

Neha Garg [a], Simo Pekkinen [a], Eduardo Martínez González [c], Rodrigo Serna-Guerrero [b], Pekka Peljo [c], Annukka Santasalo-Aarnio* [a]

When we mixed Hexacyanoferrate (II) and Hexacyanoferrite (III) together, a deep blue pigment Prussian blue salts is produced because of the oxidation of ferrous ferrocyanide Ferric Ferrocyanide complex).

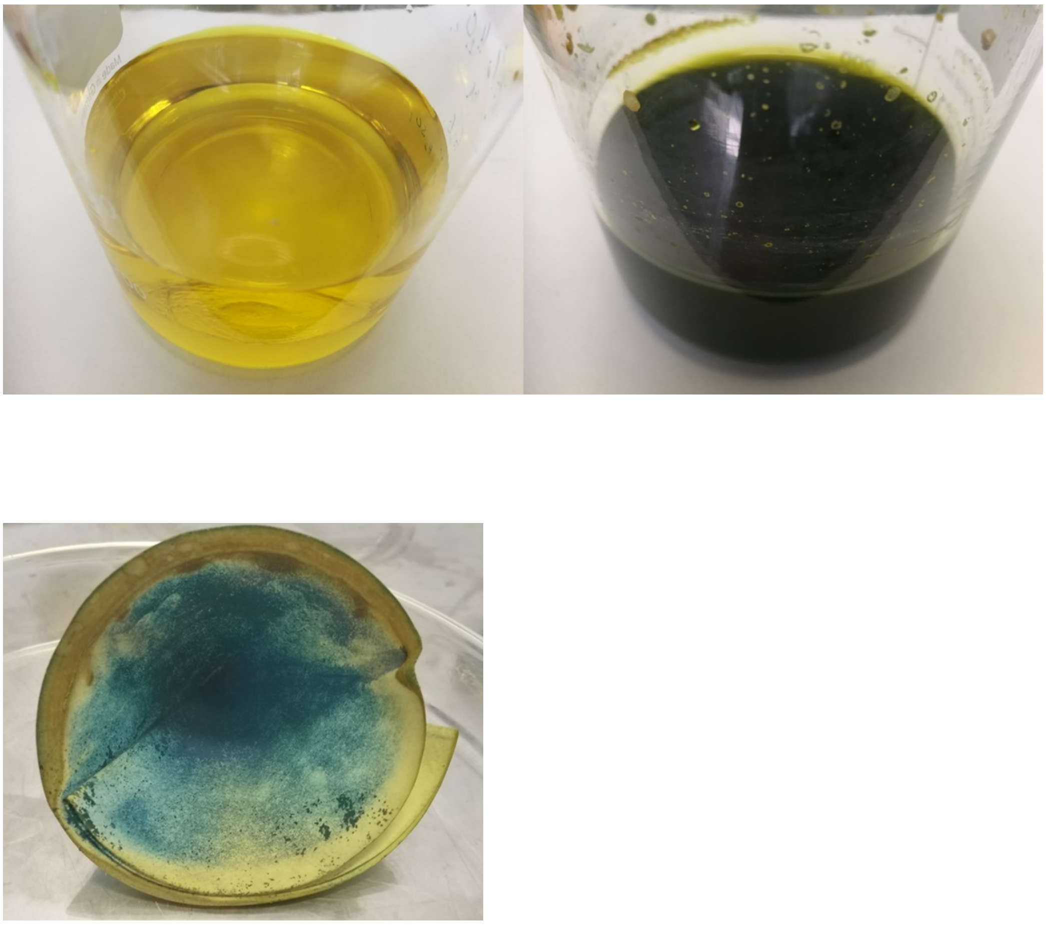

Figure S1: (a) Fe (II) and Fe (III)salt solution mixtures, (b) 96 h after addition of Biltema battery into the solution (c) Persian blue precipitate on dried filter from the solution after discharge

Finite element simulations

The simulation was performed in 2D axis symmetry, with the 6.5 cm long cylindrical battery with a radius of 0.9 cm immerced in the middle of a cylindrical container of electrolyte (radius of 12 cm and height of 15 cm). The radius of the terminal of the battery is 0.5 cm. The geometry is described in Fig. S1:

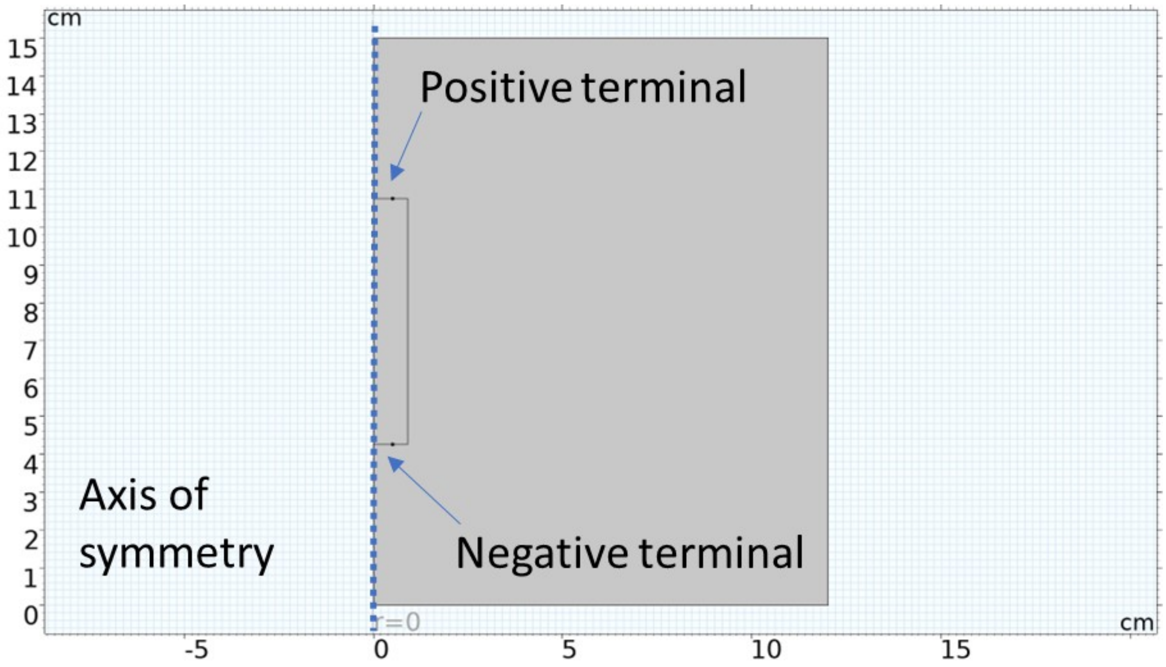

Figure S2: The simulation geometry.

The other parameters are listed in the table below:

Table S1: Simulation parameters

Parameter	Value
Diffusion coefficient of Fe(II) and Fe(III) species in electrolyte	7×10^{-6} cm^2 s^{-1}
Diffusion coefficient of H^+ in electrolyte	93×10^{-6} cm^2 s^{-1}
Diffusion coefficient of OH^- in electrolyte	50×10^{-6} cm^2 s^{-1}
Diffusion coefficient of O_2 in electrolyte	20×10^{-6} cm^2 s^{-1}
Diffusion coefficient of H_2 in electrolyte	46×10^{-6} cm^2 s^{-1}
Concentration of Fe(II) species	132 mM

Concentration of Fe(III) species	168 mM

The equations from "Transport of Diluted Species" interface of the COMSOL Multiphysics v. 6.1 were used:

$$\frac{\partial c_i}{\partial t} + \nabla \cdot \mathbf{N}_i = R_i \quad (1)$$

$$\mathbf{N}_i = -D_i \nabla c_i \quad (2)$$

where c_i is the concentration of the species i in electrolyte, $\mathbf{N}_i$ is the flux of species i, R is reaction term (here = 0), and D_i is the diffusion coefficient of species i. Considered species are Fe(III), Fe(II), H^+, OH^-, H_2 and O_2 dissolved in the electrolyte. The iron species are oxidized or reduced at the positive or negative terminals at the mass transfer limited rates. This was realized by setting concentrations of Fe(II) to 0 and Fe(III) to 300 mM at the positive terminal, and to 300 mM and 0 mM at the negative terminal. The current going to gas evolution reactions was done by substracting the mass transfer limited currents of Fe(III) reduction and Fe(II) oxidation from the experimental current in Fig. 7A. This current has to pass through both terminals. The resulting current was converted to current density by dividing with the area of the terminal, i_{side}. On the positive terminal oxygen evolution takes place at the flux of $i_{side}/F/4$ where the F is the Faraday constant and 4 comes from the stoichiometry of the oxygen evolution reaction requiring 4 electrons per one oxygen. Similarly, flux of i_{side}/F of protons is generated. On the negative terminal hydrogen is generated at the flux of $i_{side}/F/2$ and OH^- at the flux of i_{side}/F. At all the other boundaries no flux bondary condition is employed for all the species. Water dissociation reaction could be considered by this makes the model computationally very unstable. Instead, the pH was estimated by the following expression: if(c_H>c_OH,-log10((abs(c_H-c_OH)+1e-7[M])/1[M]),14+log10((abs(c_H-c_OH)+1e-7[M])/1[M])) in COMSOL.

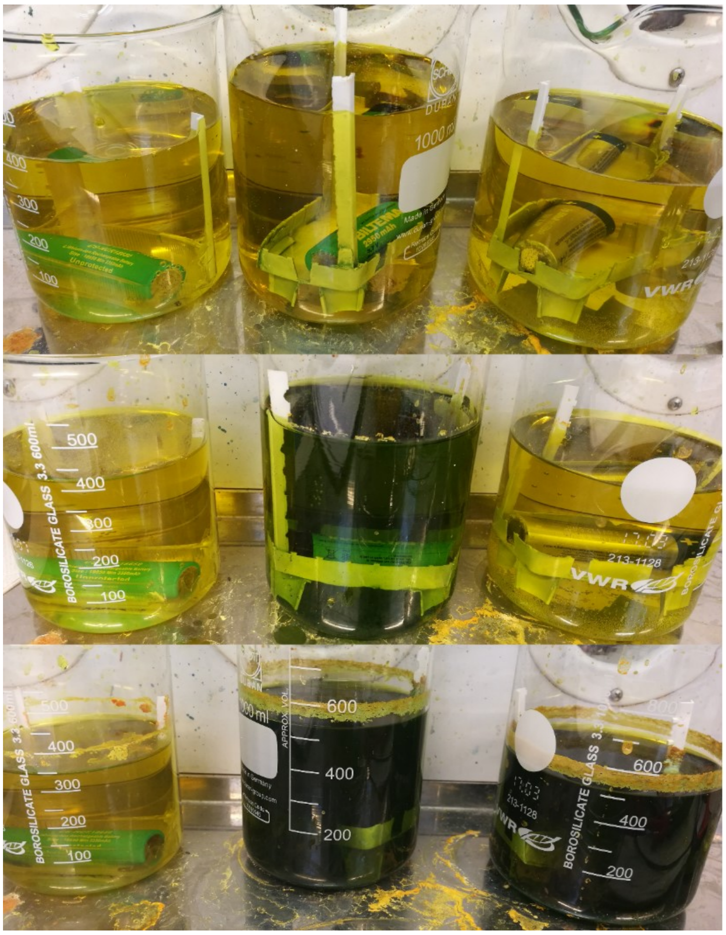

Figure S3: Three different batteries Panasonic battery, Biltema battery, and Ansmann battery respectively after discharging at (a) the same day of experiments (b) after 100 h and (c) after 250 h in redox electrolyte containing 5 wt. % electrolyte of potassium hexacyanoferrate and potassium hexacyanoferrite

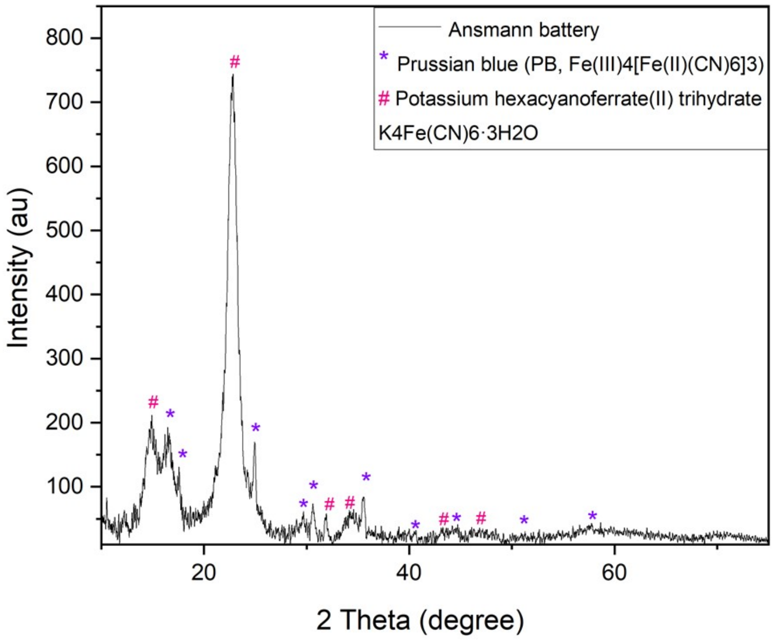

Figure S4: Powder X-ray diffraction pattern of the dried precipitate obtained from the Ansmann battery's discharging solution
